# Supplementary material for: Anticipatory and pre-planned actions: A comparison between young soccer players and swimmers
Source: PLoS One. 2021 Apr 7;16(4):e0249635. doi: 10.1371/journal.pone.0249635 (PMC8026046; doi:10.1371/journal.pone.0249635)
Supplement: S1 Table — (PDF) [file pone.0249635.s001.pdf]

Descriptives

Descriptives

	Time lag soccer	Time lag swimmer	Impulse soccer	Impulse Swimmer
N	8	8	8	8
Missing	0	0	0	0
Mean	0.108	0.118	2.34	2.16
Median	0.105	0.110	2.01	2.12
Standard deviation	0.0328	0.0231	1.39	1.01
Minimum	0.0700	0.0900	0.720	1.13
Maximum	0.150	0.150	4.38	4.23
Skewness	0.101	0.311	0.302	1.28
Std. error skewness	0.752	0.752	0.752	0.752
Kurtosis	-1.95	-1.94	-1.65	1.93
Std. error kurtosis	1.48	1.48	1.48	1.48
Shapiro-Wilk W	0.882	0.869	0.914	0.880
Shapiro-Wilk p	0.198	0.148	0.384	0.187
