# Supplementary material for: Anticipatory and pre-planned actions: A comparison between young soccer players and swimmers
Source: PLoS One. 2021 Apr 7;16(4):e0249635. doi: 10.1371/journal.pone.0249635 (PMC8026046; doi:10.1371/journal.pone.0249635)
Supplement: Attachment — Submitted filename: ResponsetoReviewers_1703.docx [file pone.0249635.s004.docx]

**Dear Editor**

**Here find below the corrections as suggested**

**Sincerely,**

**Paola Cesari**

**Reviewer #1**

Dear Authors,

Thank you for the opportunity to revise your Manuscript.

I appreciate your changes made on the paper.

I do not have any major Comments.

ANSWER. Thank you very much for your positive comment.

**Minor Comments**

Line 247 - 248. I think there is a typo error. P values should be p > 0.05 as no significant differences were detected in between the two groups in mean age, height, weight and years of experience.

ANSWER. This was a typing mistake: sorry about this.

Please add a note to (r) in Table 2 as Effect size. It may be misleading as later r is the correlation coefficient.

ANSWER. The integration has been made as suggested.
